# Association Between Borderline Personality Traits and Addictive Features of Non-Suicidal Self-Injury in Adolescent Patients With Depressive Disorder

**DOI:** 10.62641/aep.v54i2.2159

**Published:** 2026-04-15

**Authors:** Juan Wang, Qilin Zhang, Suocheng Nie, Youshan Wu

**Affiliations:** ^1^Department of Psychiatry, Jingzhou Rongjun Special Care Hospital (Jingzhou Mental Health Center), 434000 Jingzhou, Hubei, China; ^2^Department of Pharmacy, Jingzhou Rongjun Special Care Hospital (Jingzhou Mental Health Center), 434000 Jingzhou, Hubei, China

**Keywords:** borderline personality traits, adolescent, depressive disorder, non-suicidal self-injury

## Abstract

**Objective:**

This study aimed to investigate the association between borderline personality traits and addictive features of non-suicidal self-injury (NSSI) in adolescent patients with depressive disorder, identify independent risk factors for NSSI addictive features and provide evidence for clinical intervention.

**Methods:**

A cross-sectional study design was utilised. A total of 320 adolescent patients with depressive disorder (aged 12–18 years, mean ± SD: 15.82 ± 1.74 years; 238 females, 74.38%) admitted to Jingzhou Mental Health Center between January 2024 and October 2025 were enrolled. Assessments were conducted using the 17-item Hamilton Depression Rating Scale, the Borderline Personality Features Scale for Children (BPFS-C), the Adolescent Self-Rating Life Events Checklist (ASLEC), the 20-item Toronto Alexithymia Scale, the Family APGAR Index and the Addiction Subscale of the Ottawa Self-Injury Inventory (OSI-AS). Pearson correlation analysis was conducted to explore the association between borderline personality traits and NSSI addictive features. Binary logistic regression analysis was performed to identify independent influencing factors for NSSI addictive features.

**Results:**

Based on the presence or absence of NSSI addictive features, the 201 patients in the NSSI group were further divided into an addictive NSSI subgroup (n = 111) and a non-addictive NSSI subgroup (n = 90). The OSI-AS score was significantly higher in the addictive NSSI subgroup than in the non-addictive subgroup (*p* < 0.001). The total BPFS-C score and its subscale scores showed significant positive correlations with NSSI addictive features (*p* < 0.001). Binary logistic regression analysis revealed that, after adjusting for confounders such as age, gender, severity of depression and family function, the total BPFS-C score (odds ratio [OR] = 1.116, 95% confidence interval [CI]: 1.077–1.157, *p* < 0.001) and the total ASLEC score (OR = 1.051, 95% CI: 1.021–1.082, *p* = 0.001) were independent risk factors for NSSI addictive features. The overall prediction accuracy of this model was 84.7%.

**Conclusion:**

Borderline personality traits are an independent risk factor for NSSI addictive features in adolescent patients with depressive disorder and are closely associated with the severity of addictive NSSI. In clinical practice, screening for borderline personality traits should be implemented for adolescents with depression and NSSI. Early psychological interventions targeting core features such as affective instability and impulsivity should be conducted to reduce the risk of NSSI addiction.

## Introduction

Adolescence represents a critical period for neuropsychological development, and its associated mental health issues have become a key research focus in global public health [1]. The incidence of non-suicidal self-injury (NSSI) within the clinical presentation of depressive disorder is notably high, reaching up to 62.8%. This behaviour, defined as the deliberate damage to one’s own body tissue without suicidal intent, leads to physical injuries, such as skin infections and scar formation, and increases the subsequent risk of suicide attempts in adolescents by 3.2-fold, thereby posing a significant threat to their lives [2, 3]. Existing research has confirmed that exposure to negative life events (e.g., academic stress and family conflict), dysfunctional family environments and alexithymia are significant risk factors for NSSI [4, 5]. However, the influence of personality traits, as a consistent long-term psychological basis, on the mechanisms underlying NSSI has not yet been fully elucidated. Therefore, an in-depth exploration of the mechanisms influencing NSSI holds significant clinical and preventive value.

Borderline personality traits, characterised by affective instability, identity disturbance, negative interpersonal relationships and impulsivity, are observed in approximately 15%–20% of adolescent populations [6]. The core deficiency of these traits lies in compromised emotional regulation ability. Individuals with prominent traits often fail to process negative emotions such as anxiety and anger through constructive strategies, resorting instead to self-injurious behaviour to achieve temporary relief from psychological pain, thereby perpetuating a vicious cycle [7]. Although studies have observed a positive correlation between borderline personality traits and NSSI in adults with depression or in NSSI-only cohorts, neurodevelopment in adolescents is not yet mature. Borderline personality traits in this group remain in a dynamic formative stage and may exhibit different levels of expression and underlying mechanisms compared with adults. Emerging research has further elaborated these differences. Longitudinal studies suggest that adolescent borderline personality traits are primarily characterised by impulsivity and emotional instability, demonstrating lower stability than the more persistent manifestations observed in adulthood [8]. This developmental context suggests that adolescents with borderline traits may be more inclined to use immediate, tangible coping strategies (e.g., NSSI) when distressed compared with their counterparts. By contrast, adults with similar traits may exhibit established patterns of dysfunction, often resulting in chronic interpersonal difficulties [9]. Liu *et al*. [10] confirmed a significant association between borderline personality features and NSSI, specifically among adolescents. Despite these preliminary findings, the specific mechanisms by which developmental stage influences the relationship between borderline traits and NSSI remain understudied, particularly in adolescents with comorbid depressive disorder, where depressive symptoms may further amplify age-specific vulnerability.

Moreover, depressive disorder, a prevalent comorbidity of NSSI, induces symptoms such as low mood and anhedonia, which may interact with borderline personality traits to intensify the frequency and addictive nature of NSSI [11].

Addictive features of NSSI behaviours (e.g., compulsive engagement, tolerance and difficulty quitting) are associated with severe psychological impairment and elevated recurrence risk, complicating clinical intervention efforts. The relationship between borderline personality traits and NSSI addictive features in adolescents with depressive disorder remains understudied, and the independent risk factors for NSSI addiction in this population have not been clarified. Therefore, this study focused on NSSI addictive features to explore their association with borderline personality traits, aiming to provide targeted evidence for intervention.

## Materials and Methods

### Study Design and Participants

This cross-sectional study was conducted in the psychiatric outpatient and inpatient departments of Jingzhou Mental Health Center from January 2024 to October 2025. The sample size was estimated using G*Power 3.1 software (Heinrich-Heine-Universität Düsseldorf, Düsseldorf, Germany). On the basis of a hypothesised medium effect size for the association between borderline personality traits and NSSI, supported by prior research demonstrating a significant correlation [12], and establishing α = 0.05 and power (1-β) = 0.80, the minimum required sample size was calculated to be 47. During the study period, a total of 336 patients were initially screened for eligibility based on the inclusion and exclusion criteria. Among them, 16 patients were excluded due to withdrawal of consent (n = 8), incomplete questionnaires with ≥10% missing items (n = 5) and concurrent participation in other clinical trials (n = 3). The attrition rate was calculated as (16/336) × 100% = 4.76%. A total of 320 eligible adolescent patients with depressive disorder were consecutively enrolled. Consecutive enrolment was defined as sequentially recruiting all patients who met the study’s inclusion and exclusion criteria during the study period (January 2024–October 2025) in the order of their clinical visits, without selective exclusion based on non-study-related factors. Their ages ranged from 12 years to 18 years, with a mean (SD) of 15.82 (±1.74) years, including 238 females (74.38%).

Diagnoses for all participants were independently confirmed by two psychiatrists, each holding the title of associate chief physician or higher with over five years of clinical experience, according to the diagnostic criteria for depressive disorder outlined in the Diagnostic and Statistical Manual of Mental Disorders, Fifth Edition (DSM-5) [13]. The two psychiatrists conducted independent diagnostic evaluations of each patient based on DSM-5 criteria; in cases of diagnostic disagreement (occurring in 3.4% of patients), a third senior psychiatrist (with 10+ years of clinical experience) was consulted to reach a consensus. Based on the presence or absence of NSSI, patients were divided into an NSSI group (n = 201, 62.81%) and a non-NSSI group (n = 119, 37.19%). For this study, NSSI was defined as ‘the deliberate, direct destruction of one’s own body tissue without suicidal intent’ and required meeting both of the following criteria: (1) a positive response indicating at least one episode of self-injury in the past 6 months, as assessed by the Ottawa Self-Injury Inventory (OSI) [14] and used as an operational criterion for recent NSSI in this study; and (2) confirmation via a structured clinical interview (conducted by a trained psychotherapist) of the absence of suicidal motivation, and the self-injurious behaviour had resulted in tissue damage such as skin breaks and bruises (e.g., cutting, scratching and hitting).

Inclusion Criteria: The inclusion criteria were as follows: (1) aged 12–18 years; (2) met DSM-5 diagnostic criteria for depressive disorder; depressive symptom severity was assessed using the 17-item Hamilton Depression Rating Scale (HAMD-17), with a total score range of 0–54 points, and a score ≥8 points was used to confirm the presence of depressive symptoms [15]; (3) provided written informed consent from the patient and their guardian (for those under 16 years old); (4) possessed basic reading comprehension skills to complete the questionnaires independently or with guardian assistance (guardians were only permitted to read items aloud without influencing answer choices); and (5) no history of major physical illnesses (e.g., severe cardiovascular/cerebrovascular diseases and neurological diseases) in the past month.

Exclusion Criteria: The exclusion criteria were as follows: (1) comorbid with other major mental disorders including schizophrenia, bipolar disorder, autism spectrum disorder, schizoaffective disorder and severe obsessive-compulsive disorder (excluding common comorbid anxiety disorders and post-traumatic stress disorder, which were not excluded due to their high comorbidity with adolescent depressive disorder and consistent with clinical research practice for this population); (2) a history of suicide attempts or the presence of severe suicidal ideation at the time of assessment (defined by a score ≥6 on the Beck Scale for Suicide Ideation, which has been identified as the optimal cut-off value for predicting future suicidal behavior with the highest classification accuracy [16]); (3) intellectual disability (Full-Scale IQ <70 on the Wechsler Intelligence Scale for Children, Fourth Edition (WISC-IV) [17]) or significant cognitive impairment; (4) concurrent participation in other psychological intervention or pharmacological clinical trials; and (5) withdrawal from the study or questionnaire responses with >10% missing items.

The study protocol was approved by the hospital’s Ethics Committee of Jingzhou Mental Health Center (Approval No.: LL20240105). All procedures were performed in accordance with the principles of the Declaration of Helsinki. Prior to study participation, detailed information about the study (including purpose, assessment procedures, data collection methods, potential risks, benefits and data confidentiality measures) was provided to each patient and their legal guardian (for minors aged <16 years). All participants and guardians were informed that participation was voluntary, and they had the right to withdraw from the study at any time without any adverse influence on their clinical treatment. Written informed consent was obtained from all participants (and their guardians for minors) after they confirmed understanding of all study-related information.

### Assessment Instruments

#### 17-item Hamilton Depression Rating Scale (HAMD-17) 

Evaluating the severity of depression symptoms in adolescents involves 17 items that cover aspects such as depressive emotions, sleep disorders and physical symptoms [18]. Most items use a 5-level rating system, ranging from 0 to 4 points, although a few items use a 3-level rating system ranging from 0 to 2 points. The total score ranges from 0 to 54 points, with higher scores indicating more severe depression symptoms. This scale demonstrates good reliability and validity in adolescent patients with depression, as evidenced by a Cronbach’s α coefficient of 0.89 and an intraclass correlation coefficient (ICC) of 0.91.

#### Children’s Edge Characteristic Quality Scale (BPFS-C) 

The Chinese version of the Borderline Personality Inventory for Children, revised by Li Jie and Wu Mingxia [19], was used to evaluate borderline personality traits. This scale consists of 16 items and is scored on a 5-point scale (1 = never, 2 = occasionally, 3 = sometimes, 4 = frequently and 5 = always). The total score ranges from 16 points to 80 points, with higher scores indicating more prominent borderline personality traits. The scale comprises four core dimensions: emotional instability, identity confusion, negative interpersonal relationships and impulsivity. In the sample of this study, the Cronbach’s α coefficient of the scale was 0.85, indicating good internal consistency.

#### Adolescent Life Events Scale (ASLEC) 

The adolescent self-assessment life event scale developed by Liu Xianchen *et al*. [20] was used to evaluate the stress intensity caused by negative life events in the recent past. This scale consists of 27 items, covering aspects such as interpersonal relationships, academic stress, punishment, loss and health adaptation. Each item is rated on a 5-point scale based on the occurrence of the event and its psychological effect on the individual (1 = no impact, 2 = mild, 3 = moderate, 4 = severe and 5 = extremely severe). If the event does not occur, it will be scored as 1 point (no impact). The total score is the sum of the scores of each item, ranging from 27 points to 135 points. The higher the score, the greater the intensity of life event stress. The Cronbach’s α coefficient of the scale in this study was 0.83, indicating good reliability.

#### The 20-Item Toronto Alexithymia Scale (TAS-20) 

This scale was employed to assess the level of alexithymia in adolescents [21]. It contains 20 items across three dimensions: difficulty identifying feelings, difficulty describing feelings and externally oriented thinking. Items are rated on a 5-point Likert scale from 1 (strongly disagree) to 5 (strongly agree). The total score ranges from 20 to 100, with a score ≥61 indicating the presence of alexithymia [22]. The scale demonstrated a Cronbach’s α coefficient of 0.82. In the current sample, the Cronbach’s α coefficient of the TAS-20 was 0.82, which was consistent with the reliability of its Chinese version validated in Chinese adolescents with depressive disorder.

#### The Family APGAR Index 

Family functioning was evaluated using this index [23], which assesses five dimensions: adaptation, partnership, growth, affection and resolve. It consists of five items, each rated on a 3-point scale from 0 to 2 (0 = hardly ever, 2 = almost always). The total score ranges from 0 to 10. A score of 0–3 suggests severe family dysfunction, 4–6 suggests moderate dysfunction and 7–10 suggests good family function. In the present sample, the Cronbach’s α coefficient of the Family APGAR Index was 0.79, indicating acceptable internal consistency for a brief scale.

#### Ottawa Self Injury Questionnaire (OSI)

This questionnaire is used to comprehensively evaluate the behavioural characteristics of NSSI, including frequency, mode, addiction and functional motivation [24]. The Addictive Inventory consists of 7 items, adapted from the DSM-IV-TR Substance Dependence Criteria, used to evaluate the addictive characteristics of self-injurious behaviour. Each item is rated on a five-point scale of 0–4 (0 = never, 4 = always), with a total score of 0–28. When there are ≥3 items with a score of ≥2, it is defined as having NSSI addictive characteristics. This study adopted the Chinese-adapted version of the OSI, which was adjusted for cultural compatibility with Chinese adolescents. In the current sample, the Cronbach’s α coefficient for the total scale was 0.89, whereas that for the Addiction Subscale was 0.87, both indicating excellent internal consistency. The adapted version retains the original scale’s structural validity, as supported by preliminary validation in Chinese clinical samples.

### Data Collection

All assessors (two psychiatrists and three psychotherapists) received standardised training covering scale administration protocols, structured interview techniques and NSSI determination criteria. Those who passed the post-training assessment (achieving ≥90% accuracy in simulated case evaluations) were deemed eligible to participate in data collection.

Data collection was conducted in a quiet research-specific consultation room adhering to the following procedure: (1) First, the assessor explained the study purpose, procedures and data confidentiality principles to the patient and guardian and then obtained written informed consent. (2) Basic information was collected using a general information questionnaire, and NSSI behaviour (including time of occurrence, frequency and methods) was confirmed via a structured interview. (3) Patients were guided to complete the HAMD-17 (rated by the physician), BPFS-C, ASLEC, TAS-20, Family APGAR and OSI (all self-report; for participants under 14 years old, guardians filled out the forms based on the patient’s verbal responses). (4) Upon completion, the assessor reviewed the questionnaires onsite. If missing items were <5%, they were completed immediately through follow-up questioning. Questionnaires with ≥10% missing items were deemed invalid and excluded, and the research team recruited new eligible patients to maintain the target sample size.

### Statistical Analysis

Data analysis was performed using SPSS 26.0 statistical software (IBM Corp., Armonk, New York, USA). The normality of continuous variables was tested using the Shapiro–Wilk test, and the homogeneity of variance was tested using Levene’s test. If continuous variables did not meet the assumption of normal distribution (Shapiro–Wilk test, *p* < 0.05), they were presented as median (interquartile range) [M (Q1, Q3)], and group comparisons were performed using the Mann–Whitney U test (for two independent groups). For correlations involving non-normally distributed continuous variables, Spearman rank correlation analysis was adopted. Categorical variables were consistently expressed as frequency (percentage) [n (%)], with group comparisons using the χ^2^ test regardless of data distribution. Pearson correlation analysis was employed to explore the associations between the total and subscale scores of the BPFS-C and the characteristics of NSSI behaviour. Multivariate binary logistic regression analysis was conducted to identify independent influencing factors for NSSI behaviour. The forward stepwise method (entry criterion: *p* < 0.05; removal criterion: *p* > 0.10) was adopted for variable selection, based on the Akaike Information Criterion to optimise model fit. Prior to regression analysis, multicollinearity among independent variables was tested using the variance inflation factor (VIF). The results showed that the VIF values of all variables (HAMD-17 score: 1.52; BPFS-C total score: 1.68; ASLEC total score: 1.45; Family APGAR: 1.39; age: 1.08; and gender: 1.05) were <10, indicating no severe multicollinearity in the model. The fit of the regression model was assessed using Nagelkerke’s R^2^, and its predictive performance was evaluated by the overall correct classification rate. A two-tailed *p* value < 0.05 was considered statistically significant.

## Results

### Demographic and Clinical Characteristics

No significant differences were observed between the two groups regarding age or gender distribution (*p* > 0.05). The NSSI group demonstrated significantly higher scores on the HAMD-17, BPFS-C, ASLEC and OSI compared with the non-NSSI group (*p* < 0.001) (Table 1).

**Table 1.** **Comparison of demographic and clinical characteristics**.

Characteristic		NSSI group (n = 201)	Non-NSSI group (n = 119)	U/χ^2^/t	*p* value
Age (years)		15.76 ± 1.81	15.91 ± 1.62	0.745	0.457
Gender, n (%)					
	Female	153 (76.12)	85 (71.43)	0.863	0.353
	Male	48 (23.88)	34 (28.57)		
Educational level, n (%)				0.010	0.919
	Middle school	112 (55.72)	67 (56.30)		
	High school or equivalent	89 (44.28)	52 (43.70)		
Disease duration (months)		7.86 ± 3.21	9.12 ± 3.05	3.457	0.001
HAMD-17 score		24.35 ± 5.62	19.41 ± 4.83	7.997	<0.001
BPFS-C total		52.18 ± 8.75	38.25 ± 7.36	14.578	<0.001
ASLEC total		45.00 (38.00, 56.00)	37.00 (30.00, 46.00)	6.161	<0.001
OSI Addiction Subscale score		18.20 ± 3.53	7.15 ± 2.80	29.143	<0.001
TAS-20 total		62.25 ± 9.17	55.31 ± 8.42	8962.5	<0.001
Family APGAR		5.00 (4.00, 7.00)	7.00 (6.00, 8.00)	7641.0	<0.001

**Note**: HAMD-17, 17-item Hamilton Depression Rating Scale; BPFS-C, Borderline Personality Features Scale for Children; ASLEC, Adolescent Self-Rating Life Events Checklist; OSI, Ottawa Self-Injury Inventory; TAS-20, Addiction Subscale of the Ottawa Self-Injury Inventory. Data are presented as mean ± standard deviation or frequency (percentage).

### Association Between Borderline Personality Traits and NSSI Characteristics

Within the NSSI group, the correlation between borderline personality traits (BPFS-C scores) and the characteristics of NSSI behaviour was further analysed. The total BPFS-C score and its subscale scores all showed significant positive correlations with the OSI Addiction Subscale (*p* < 0.001). Additionally, the total BPFS-C score and its four subscales (emotional instability, identity disturbance, negative interpersonal relationships and impulsivity) were significantly positively correlated with the emotional regulation function of NSSI (*p* < 0.001). Among them, the emotional instability dimension of BPFS-C had the highest correlation with the emotional regulation function of NSSI (*r* = 0.512, 95% confidence interval [CI]: 0.425–0.591), whereas the impulsivity dimension showed the strongest correlation with NSSI addictive features (r = 0.538, 95% CI: 0.451–0.615) (Table 2).

**Table 2.** **Correlation analysis between borderline personality traits and NSSI behaviour characteristics**.

Variable	BPFS-C total		Emotional instability		Identity disorder		Negative relationship		Impulsive	
	*r*	*p*	*r*	*p*	*r*	*p*	*r*	*p*	*r*	*p*
OSI addiction	0.524 (0.436–0.603)	<0.001	0.471 (0.377–0.555)	<0.001	0.493 (0.401–0.575)	<0.001	0.438 (0.340–0.526)	<0.001	0.538 (0.451–0.615)	<0.001
NSSI emotion regulation function	0.501 (0.412–0.582)	<0.001	0.512 (0.425–0.591)	<0.001	0.423 (0.324–0.514)	<0.001	0.396 (0.295–0.488)	<0.001	0.445 (0.348–0.533)	<0.001

**Note**: NSSI addictive features were assessed by the OSI Addiction Subscale; *r* = Pearson correlation coefficient.

### Analysis of Addictive Features of NSSI and Related Factors

Within the NSSI group, 55.22% (111/201) of patients exhibited addictive features in their NSSI behaviour, with the impulsivity dimension showing the strongest correlation with addictive features (r = 0.538). Univariate analysis revealed that the addictive NSSI subgroup had significantly higher scores on the HAMD-17, total BPFS-C, total ASLEC and total TAS-20 compared with the non-addictive subgroup (*p* < 0.001). Conversely, their level of family support (Family APGAR) was significantly lower than that of the non-addictive subgroup (*p* < 0.001) (Table 3).

**Table 3.** **Comparison of characteristics between the addictive and non-addictive NSSI subgroups**.

Characteristic	Addictive NSSI (n = 111)	Non-addictive NSSI (n = 90)	*T *value	*p* value
HAMD-17 score	26.89 ± 5.01	21.12 ± 4.75	8.309	<0.001
BPFS-C total	57.25 ± 7.18	45.62 ± 6.94	11.591	<0.001
ASLEC total	50.00 (42.00, 58.00)	40.00 (32.00, 48.00)	6.215	<0.001
TAS-20 total	66.78 ± 8.95	56.95 ± 9.01	7.720	<0.001
Family APGAR	5.00 (4.00, 6.00)	7.00 (6.00, 8.00)	7.328	<0.001

**Note**: NSSI, non-suicidal self-injury; HAMD-17, 17-item Hamilton Depression Rating Scale; BPFS-C, Borderline Personality Features Scale for Children; ASLEC, Adolescent Self-Rating Life Events Checklist; TAS-20, 20-item Toronto Alexithymia Scale. Data are presented as mean ± standard deviation.

### Logistic Regression Analysis of Influencing Factors for NSSI Behaviour

To clarify the independent predictive effect of borderline personality traits on NSSI behaviour, we assessed the presence or absence of NSSI addictive features (1 = yes, 0 = no). After adjusting for confounding factors including age, gender, severity of depression and family function, the HAMD-17 score (odds ratio [OR] = 1.094, 95% CI: 1.031–1.161, *p* = 0.003), total BPFS-C score (OR = 1.116, 95% CI: 1.077–1.157, *p* < 0.001) and total ASLEC score (OR = 1.051, 95% CI: 1.021–1.082, *p* = 0.001) were identified as independent risk factors for NSSI addictive features. The overall prediction accuracy of this model was 84.7% (Table 4).

**Table 4.** **Binary logistic regression analysis of influencing factors for NSSI behaviour**.

Variable	B	SE	Waldχ^2^	*p* value	OR value	95% CI
Age	−0.050	0.060	0.694	0.405	0.951	0.846–1.069
Gender (female)	0.320	0.280	1.306	0.253	1.377	0.796–2.382
HAMD-17 score	0.089	0.030	8.827	0.003	1.094	1.031–1.161
BPFS-C total	0.109	0.019	32.815	<0.001	1.116	1.077–1.157
ASLEC total	0.049	0.015	10.671	0.001	1.051	1.021–1.082
Family APGAR	–0.249	0.069	13.012	<0.001	0.779	0.679–0.893

**Note**: The dependent variable was the presence or absence of NSSI behaviour (1 = yes, 0 = no). Overall model χ^2^ = 98.632, *p* < 0.001, Nagelkerke R^2^ = 0.402. HAMD-17, 17-item Hamilton Depression Rating Scale; BPFS-C, Borderline Personality Features Scale for Children; ASLEC, Adolescent Self-Rating Life Events Checklist. Dependent variable: Presence or absence of NSSI addictive features (defined by ≥3 items with score ≥2 on the OSI Addiction Subscale).

### Gender Interaction and Stratified Analysis

The interaction term of the BPFS-C total score and gender in the multivariate logistic regression model was not statistically significant (OR = 1.023, 95% CI: 0.958–1.093, *p* = 0.487), indicating that the association between borderline personality traits and NSSI did not differ by gender. Stratified analyses showed that in female adolescents (n = 238), the BPFS-C total score was associated with NSSI (OR = 1.109, 95% CI: 1.065–1.155, *p* < 0.001). In male adolescents (n = 82), the BPFS-C total score was also associated with NSSI (OR = 1.132, 95% CI: 1.049–1.222, *p* = 0.002), exhibiting a consistent direction and similar magnitude of association between genders.

## Discussion

This study investigated the association between borderline personality traits and NSSI in 320 adolescent patients diagnosed with depressive disorder according to DSM-5 criteria. The results indicated an NSSI incidence rate of 62.81%. Borderline personality traits (total BPFS-C score) were positively correlated with NSSI frequency, number of methods and addictive features. Even after adjusting for confounding factors such as age, gender and severity of depression, these traits remained independently associated with NSSI occurrence; nevertheless, the cross-sectional design prevents causal inference. This confirms the importance of systematically assessing borderline personality traits to determine NSSI risk among adolescents with depression.

The findings of this study showed that patients in the NSSI group had higher total and subscale scores on the BPFS-C compared with those in the non-NSSI group. This suggested that the influence of borderline personality traits on NSSI may stem from their core deficiencies in emotional regulation and interpersonal dysfunction. Borderline personality traits are characterised by emotional instability, identity confusion, negative interpersonal relationships and impulsivity, which may be associated with NSSI behaviour through multiple pathways [25]. Affective instability, the BPFS-C dimension most strongly correlated with the emotional regulation function of NSSI, reflects functional abnormalities in the neural circuits regulating emotion in these patients. When confronted with negative emotions, these adolescents exhibit weakened cognitive control functions in the prefrontal cortex and heightened activation of emotional centres such as the amygdala. This impairs their ability to use adaptive strategies for emotional regulation, leading them to resort to the physical pain of NSSI as a means to alleviate psychological distress, thereby reinforcing the cycle of self-injurious behaviour [26, 27]. The correlation between identity disruption and NSSI frequency suggests that a vague self-concept plays a role in self-injurious behaviour. The uncertainty of self-identity and inadequate social relationships in adolescence may further exacerbate psychological vulnerability, rendering NSSI a means to validate self-existence or manage interpersonal stress [28]. NSSI may serve as an existential tool, using the tangible sensation of bodily injury to confirm self-existence or employing the physiological responses following self-harm to counteract emotional numbness. Additionally, the correlation between the negative relationships dimension and the social influence function of NSSI indicates that social difficulties such as interpersonal conflict and fear of abandonment may lead to NSSI being used as a tool to express distress, seek attention or control relationships, particularly in patients with low family support. This inference is strongly supported by the correlation results of this study: the emotional instability dimension of BPFS-C showed the highest correlation with the emotional regulation function of NSSI, which confirmed that affective instability (core to borderline personality traits) is the key link between these traits and NSSI’s emotional regulation function. Additionally, the impulsivity dimension had the strongest correlation with NSSI addictive features, verifying that impairments in behavioural control directly facilitate the formation of addictive self-injury patterns. The identity disturbance dimension was positively correlated with NSSI frequency, indicating that a vague self-concept in adolescents with borderline traits may enhance psychological vulnerability, rendering NSSI a mechanism for managing identity confusion.

In this study, 55.22% of patients with NSSI exhibited addictive features. The addictive NSSI subgroup had significantly higher scores on the HAMD-17 and total BPFS-C and significantly lower levels of family support (Family APGAR) compared with the non-addictive subgroup. The Family APGAR Index evaluates current family functioning (without a specified retrospective time window, reflecting the ongoing family support level at the time of assessment). The impulsivity characteristic of borderline personality traits may reduce an individual’s control over self-injurious behaviour, whereas impaired family function weakens the protective influence of social support against negative emotions. Coupled with severe depressive symptoms amplifying emotional pain, these three factors are synergistically associated with the transition of NSSI from an occasional behaviour to an addictive pattern [29]. Furthermore, when NSSI occurs, the transient emotional relief brought by the release of endogenous opioids, combined with the dopamine-mediated reward effect, creates a cycle similar to substance addiction. With behavioural repetition, individuals may develop tolerance, requiring increased frequency or intensity of self-injury to achieve the same degree of emotional release, ultimately leading to NSSI addiction [30]. This study revealed that the addictive subgroup had significantly higher total ASLEC scores than the non-addictive subgroup, indicating that chronic or severe life event stress may enhance an individual’s dependence on immediate relief mechanisms via dysregulation of the hypothalamic–pituitary–adrenal axis function [31]. Simultaneously, low family functioning reduces the protective mechanism of social support, making patients susceptible to a vicious cycle of stress and self-injury. The significantly higher TAS-20 scores in the addictive subgroup relative to the non-addictive subgroup suggest that difficulties in identifying and expressing emotions may hinder patients from seeking help through verbal communication, thereby increasing the risk of NSSI occurrence.

The results of the multivariate binary logistic regression analysis in this study identified the total BPFS-C score and the total ASLEC score as independent risk factors for NSSI addictive features. Given that addictive features represent a severe and persistent pattern within NSSI behaviours, these findings highlight specific risk factors associated with the pathological and treatment-resistant variants of self-injury. This suggests that borderline personality traits may be a more stable indicator of the statistical association with the severity and addictive potential of NSSI compared with depressive symptoms. Depressive symptoms often fluctuate with treatment and environmental changes, whereas personality traits, as relatively stable psychological constructs, may exert a more persistent influence [32]. The regression results showed that borderline personality traits (OR = 1.116) had a stronger predictive effect on NSSI than negative life events (OR = 1.051), which can be explained by three aspects. First, as relatively stable psychological constructs, borderline personality traits are anchored in long-term developmental processes and neurobiological foundations, whereas negative life events are temporary external stressors; this stability makes traits a more reliable predictor than life events. Second, neurobiologically, this study’s findings were aligned with prior neuroimaging evidence [33, 34] indicating that adolescents with significant borderline traits exhibit abnormal functional connectivity between the prefrontal cortex (responsible for emotional regulation and impulse control) and the amygdala (emotional processing centre), which constitutes a biological basis for sustained vulnerability to NSSI. By contrast, negative life events primarily act as external triggers without altering the intrinsic neuropsychological vulnerability. Third, borderline personality traits synergise with depressive symptoms and family dysfunction: the traits themselves involve deficits in emotion regulation and interpersonal adaptation, which amplify the impact of negative life events, whereas negative life events alone do not produce such synergistic effects on various psychological and behavioural dimensions. The ASLEC assesses the severity of negative life event stress experienced in the past 12 months, which includes the 6-month assessment period for NSSI (defined by at least one episode in the past 6 months). The sample was predominantly female (74.38%), and this gender disparity may limit the generalisability of the findings to male adolescents. To address this, we added a BPFS-C × gender interaction term and conducted stratified analyses, which revealed no significant gender difference in the association between borderline personality traits and NSSI. Nonetheless, the limited sample size of male participants (n = 82) may reduce the statistical ability to detect potential subtle gender differences, and future studies with balanced gender representation are needed to validate the current findings.

Several limitations warrant consideration. This study is a cross-sectional design, and the results only indicate an independent association between borderline personality traits and non-suicidal self-harm behaviour, without permitting causal inferences. Future longitudinal and multi-centre studies incorporating objective measures, such as neurobiological markers or ecological momentary assessment, will elucidate the temporal and mechanistic pathways linking borderline personality traits to NSSI. Nevertheless, this study provides meaningful evidence to guide early detection and intervention strategies aimed at reducing the burden of NSSI in adolescents with depression. Additionally, potential measurement bias must be recognised: several scales used in this study (e.g., ASLEC and BPFS-C) rely on self-reports, which may be influenced by recall bias and social desirability bias, potentially affecting the objectivity of measurement results. Furthermore, the study did not include key confounding factors, such as past trauma history and broad social support levels. These factors are well-documented to be associated with NSSI and may interact with borderline personality traits, which could limit the comprehensiveness of the current risk factor analysis.

## Conclusion

This study provides robust evidence that borderline personality traits are independently associated with NSSI among adolescent patients with depressive disorder, even after controlling for depression severity, negative life events and family functioning. Results indicated a clear association between these traits and key dimensions of NSSI, including frequency, methods, addictive features and emotion-regulation functions. Specifically, affective instability and impulsivity, which are core components of borderline personality traits, appear to be critical factors related to the onset and persistence of self-injurious behaviour in this vulnerable population.

From a clinical perspective, these results highlight the importance of systematically screening for borderline personality features in adolescents presenting with depressive symptoms. Early identification of such traits could facilitate targeted psychological interventions aimed at improving emotional regulation, reducing impulsivity and enhancing interpersonal effectiveness, thereby reducing the risk of NSSI. Moreover, interventions should address co-occurring factors such as high life stress and low family support, which exacerbate the risk and addictive potential of self-injury.

## Availability of Data and Materials

The data that support the findings of this cross-sectional study were collected at Jingzhou Mental Health Center and are not publicly available due to ethical restrictions protecting patient confidentiality. De-identified data may be made available from the corresponding author upon reasonable request and with permission from the relevant ethics committee.
